# A Functional Variant in the *MTOR* Promoter Modulates Its Expression and Is Associated with Renal Cell Cancer Risk

**DOI:** 10.1371/journal.pone.0050302

**Published:** 2012-11-28

**Authors:** Qiang Cao, Xiaobing Ju, Pu Li, Xiaoxin Meng, Pengfei Shao, Hongzhou Cai, Meilin Wang, Zhengdong Zhang, Chao Qin, Changjun Yin

**Affiliations:** 1 State Key Laboratory of Reproductive Medicine, Department of Urology, the First Affiliated Hospital of Nanjing Medical University, Nanjing, China; 2 Department of Molecular and Genetic Toxicology, School of Public Health, Nanjing Medical University, Nanjing, China; Sanjay Gandhi Medical Institute, India

## Abstract

**Background:**

The mTOR signaling pathway plays a crucial role in the carcinogenesis of renal cell cancer (RCC). We sought to investigate the influence of genetic variations in the mTOR pathway-related genes on the risk of RCC.

**Methods:**

We genotyped 8 potentially functional polymorphisms in *AKT1*, *AKT2*, *PTEN* and *MTOR* genes using the TaqMan method in a case-control study of 710 RCC patients and 760 cancer-free subjects. Unconditional logistic regression, adjusted for potential confounding factors, was used to assess the risk associations. We then examined the functionality of the important polymorphisms.

**Results:**

Of the 8 polymorphisms, after adjusting for multiple comparisons, we found a significant association between one variant (rs2295080) in the promoter of *MTOR* and reduced RCC risk (*P* = 0.005, OR = 0.74, 95%CI = 0.59–0.91, TG/GG *vs.* TT). Another variant (rs701848) in the 3′UTR region of *PTEN* was associated with increased RCC risk (*P* = 0.014, OR = 1.45, 95%CI = 1.08–1.96, CC vs. TT); however, the association was not significant after adjusting for multiple comparisons. Furthermore, we observed lower *MTOR* mRNA levels in the presence of the rs2295080G allele in normal renal tissues. The luciferase reporter assay showed that the rs2295080G allele significantly decreased luciferase activity. No other significant association between the selected polymorphisms and RCC risk was observed.

**Conclusions:**

Our results suggest that the functional *MTOR* promoter rs2295080 variant affects RCC susceptibility by modulating the endogenous *MTOR* expression level. The risk effects and the functional impact of the *MTOR* rs2295080 variant need further validation.

## Introduction

Renal cell cancer (RCC) is the most common type of kidney cancer, accounting for more than 80% of all malignant kidney tumors [1], [2]. Although the exact cause of RCC remains largely unknown, aberrant angiogenesis is considered a hallmark of this disease [3], [4]. In the majority of sporadic and hereditary RCC cases, the von Hippel-Lindau (VHL) tumor suppressor gene is functionally disrupted and results in constitutive activation of hypoxia-inducible factor (HIF) and subsequent induction of target genes, such as VEGF [4]. Genetic variations in angiogenesis-related genes have been suggested to influence individuals' susceptibility to RCC [5], [6], [7]. Recently, a large genome-wide association study conducted in the United States has identified an interesting variant in *EPAS1* (encoding HIF-2 alpha) as a susceptibility locus for RCC in a European population [7]. However, in the Chinese population that we evaluated, we failed to replicate the significant findings between polymorphisms in *HIF1A* [8] as well as *EPAS1* [9] and the risk of RCC, which indicates that there are differences in the genetic architecture of ethnic groups, and investigating the genetic variations in other candidate genes is still necessary. Herein, in the present study, we expanded the exploration to an important signaling pathway comprising phosphoinositide-3-kinase (PI3K), phosphatase and tensin homolog (PTEN), v-akt murine thymoma viral oncogene homolog (AKT), and mammalian target of rapamycin (mTOR).

The mTOR signaling pathway plays a crucial role in cell growth, survival, proliferation and angiogenesis [10]. PI3Ks are activated by receptor tyrosine kinases such as epidermal growth factor receptor (EGFR), vascular endothelial growth factor receptor (VEGFR) and insulin-like growth factor receptor (IGFR); the activation then results in a kinase cascade through AKT and mTOR [11]. This pathway is negatively regulated by the tumor suppressor gene *PTEN* through the dephosphorylation of phosphatidylinositol trisphosphate (PIP3) [12]. Genetic alterations of mTOR pathway-related genes, including mutations of *PI3K*, *AKT*, and *PTEN*, facilitate tumorigenesis and are common in human cancers [13], [14], [15]. The relevance of mTOR signaling in RCC is highlighted by the success in using inhibitors of mTOR (temsirolimus and everolimus) to treat patients with advanced disease [16], [17].

Single nucleotide polymorphisms (SNPs) in candidate genes have been proven to influence individuals' susceptibility to RCC [7]. In light of the critical role of the mTOR pathway in RCC, it is possible that SNPs in this pathway may play an important role in RCC development. However, no published study has yet addressed this issue. Accordingly, in the present study, we reviewed 5 core genes (*PI3KCA*, *AKT1*, *AKT2*, *PTEN*, and *MTOR*) in this pathway and analyzed 8 potentially functional SNPs in these genes and their impact on the occurrence of RCC in a Chinese population.

## Patients and Methods

### Ethics statement

The study was approved by the Institutional Review Board of the Nanjing Medical University, Nanjing, China. At recruitment, written informed consent was obtained from all participants involved in this study.

### Study population

Overall, 710 incident patients with RCC and a group of 760 cancer-free controls recruited at the First Affiliated Hospital of Nanjing Medical University, Nanjing, China between May 2004 and September 2011 were enrolled in the case-control study. The inclusion criteria of cases and controls have been described elsewhere [8]. Briefly, all of the newly diagnosed patients with histopathologically confirmed incident RCC and without prior history of other cancers or previous chemotherapy or radiotherapy were consecutively recruited without the restriction of age and sex. The disease was classified according to the World Health Organization criteria and staged according to the 2002 American Joint Committee on Cancer (AJCC) TNM classification. The controls were recruited from subjects who were seeking physical examination in the outpatient departments at the hospital and were frequency matched to the cases by age (±5 years) and sex. The cancer-free controls were genetically unrelated to the cases and had no individual history of cancer. Before recruitment, a standard questionnaire was administered through face-to-face interviews by trained interviewers to collect demographic data and related factors. Each patient donated 5 mL venous blood after providing a written informed consent. The response rate for case and control subjects was above 85%.

### SNP selection

We reviewed 5 core genes involved in the mTOR signaling pathway: *PI3KCA*, *AKT1*, *AKT2*, *PTEN* and *MTOR*. SNPs in these genes were selected based on HapMap data (http://hapmap.ncbi.nlm.nih.gov/) and dbSNP data (http://www.ncbi.nlm.nih.gov/projects/SNP/). The potentially functional polymorphisms were identified according to the following criteria: (1) located in the 5′ flanking regions, 5′ untranslated region (UTR), 3′ UTR, or coding regions with amino acid changes; (2) minor allele frequency (MAF) >5% in the Chinese population; or (3) associated with cancer risk in previous studies. According to the criteria, 8 SNPs were identified, including rs2494750 and rs2498786 in *AKT1*, rs33933140 and rs7254617 in *AKT2*, rs11202607 and rs701848 in *PTEN* as well as rs2295080 and rs2536 in *MTOR*.

### DNA extraction and genotyping

Genomic DNA was extracted from the peripheral blood by proteinase K digestion and phenol-chloroform extraction. The genotyping of these 8 SNPs was performed using predesigned TaqMan SNP Genotyping Assays (Applied Biosystems, Foster City, CA, USA) in the Laboratory of the Department of Molecular and Genetic Toxicology, Nanjing Medical University, Nanjing, China. The sequences of the primers and probes are listed in Table S1. The reaction mixture of 10 µL contained 20 ng genomic DNA, 3.5 µL of 2× TaqMan Genotyping Master Mix, 0.25 µL of the primers and probes mix and 6.25 µL of double distilled water. The amplification was performed under the following conditions: 50°C for 2 min, 95°C for 10 min followed by 45 cycles of 95°C for 15 sec, and 60°C for 1 min. Amplifications and analysis were performed in the 384-well ABI 7900HT Real Time PCR System (Applied Biosystems) following the manufacturer's instructions. SDS 2.4 software (Applied Biosystems) was used for allelic discrimination. The genotyping rates of these SNPs were all above 98%. For quality control, 4 negative controls were included in each plate and 5% of the samples were randomly selected for repeated genotyping for confirmation; and the results were 100% concordant.

### Analysis of *MTOR* mRNA expression

Eighteen surgically removed renal cancer tissues with paired paratumor renal tissues and an additional 24 paratumor renal tissues were used to analyze *MTOR* mRNA levels *in vivo*. The tissues were taken from the surgically removed samples from the patients and were immediately stored in liquid nitrogen. The RNA was isolated from about 100 mg tissue using TRIzol reagent (Invitrogen, Carlsbad, CA, USA) and reverse transcribed to single-stranded cDNA using an oligo(dT) primer and Superscript II (Invitrogen). The *MTOR* RNA level was measured by quantitative real-time reverse transcription (RT)-PCR on the ABI Prism 7900 sequence detection system (Applied Biosystems, Foster City, CA, USA). The *ACTB* was used as an internal reference gene. The primers used for *MTOR* were 5′ -TTGCTTGAGGTGCTACTG -3′ (sense) and 5′-CTGACTTGACTTGGATTCTG-3′ (antisense), and the primers for *ACTB* were 5′-TGGCACCCAGCACAATGAA-3′ (sense) and 5′-CTAAGTCATAGTCCGCCTAGAAGCA-3′ (antisense). The reaction mixture contained 0.1 M of each primer, 2×SYBR Green PCR Master Mix (TaKaRa, Berkeley, CA, USA), and 1 µL of cDNA (1∶10 dilution). The amplification was performed under the following conditions: 95°C for 30 s, and 40 cycles of 95°C for 15 s and 60°C for 30 s. Each reaction was done in triplicate.

### Construction of promoter-reporter plasmids

To construct the target *MTOR* promoter-reporter plasmids, we synthesized the DNA fragment containing either the rs2295080G allele or the T allele by amplifying the 998-bp (from −617 to 381 base relative to the transcription start site) *MTOR* promoter region using primers with restriction sites. The primers were 5′-ACTTAGAGCTCAAACAGGGATGGGGCTGGGGGAGAGGGA-3′ (forward) and 5′-ACTTAAGATCTCGAAACGTCTTTTGATGCAGTAATTCCT-3′ (reverse), and they included the SacI and NheI restriction sites. The resulting PCR products were subsequently digested with SacI and NheI and cloned into the pGL3-Basic vector (Promega, Madison, WI, USA) containing the firefly luciferase gene as a reporter. The constructs were all confirmed by DNA sequencing.

### Cell lines

The renal cell adenocarcinoma cell line (786-o), Human Embryonic Kidney 293 cells (HEK-293) and HeLa cell lines were kindly provided by Dr. Z. Zhang (Department of Molecular and Genetic Toxicology, School of Public Health, Nanjing Medical University, Nanjing, China) and were used as reported previously [18], [19].

### Transfection and luciferase reporter assays

786-o, HEK-293 and HeLa cells were seeded in 24-well culture plates. After 24 h, each well was transfected with 1 µg of each *MTOR*-reporter plasmid using Lipofectamine 2000 (Invitrogen). As an internal standard, all plasmids were cotransfected with 8 ng pRL-SV40, which contained the Renilla luciferase gene. The pGL3-Basic vector without an insert was used as a negative control. Forty-eight hours after transfections, cells were lysed with the passive lysis buffer (Promega) and assayed for luciferase activity using the Dual-Luciferase Reporter Assay System (Promega). Independent experiments were done in triplicate for each plasmid construct.

### Statistical analyses

Differences in the distributions of demographic characteristics, selected variables, and frequencies of genotypes between cases and controls were tested by the Student's t-test (for continuous variables) or χ^2^-test (for categorical variables). SNP allele frequencies in control participants were tested against departure from Hardy–Weinberg equilibrium by a goodness-of-fit χ^2^-test before further analysis. The associations between polymorphisms and risk of RCC were estimated by computing odds ratios (ORs) and 95% confidence intervals (CIs) from unconditional logistic regression analysis with the adjustment for possible confounders. We used the false discovery rate (FDR) based on the Benjamini-Hochberg method to adjust the *P* value for multiple comparisons. The associations were considered statistically significant when FDR-adjusted *P* values were less than 0.05. Differences in luciferase reporter gene expression among different promoter constructs, as well as mRNA and protein levels from renal tumor or normal tissues carrying different genotypes were evaluated by the Student's t test or one-way analysis of variance (ANOVA), and *P*<0.05 was considered to be statistically significant. All analyses were performed with the software SAS 9.1.3 (SAS Institute, Cary, NC, USA) with two-sided *P* values.

## Results

### Characteristics of RCC patients and controls

The frequency distributions of selected characteristics of the 710 cases and 760 controls are shown in Table 1. There were no significant differences between the cases and controls with regard to age, sex, BMI and drinking status (all *P*>0.05). However, there were more smokers, hypertension patients and diabetics among the cases than among the controls (*P* = 0.035, <0.001 and <0.001, respectively). Of 710 patients, 62.8% of the patients were in stage I, whereas 19.6, 7.3 and 10.3% were found to have stage II, III and IV diseases, respectively. The percent of nuclear grade from I to IV was 19.2, 48.0, 24.5 and 8.3 respectively.

**Table 1:** Distribution of selected variables between the renal cell carcinoma cases and control subjects.

Variables	Cases (n = 710)		Controls (n = 760)		*P* *
	*N*	%	*N*	%	
Age (years) (mean ± SD)	56.9±11.9		56.8±11.6		0.753
≤57	364	51.3	423	55.7	0.092
>57	346	48.7	337	44.3	
BMI (kg/m2) (mean ± SD)	24.1±2.8		23.8±3.2		0.078
<24	346	48.7	391	51.5	0.298
≥24	364	51.3	369	48.5	
Sex					
Male	454	63.9	490	64.5	0.832
Female	256	36.1	270	35.5	
Smoking status					
Never	444	62.5	515	67.8	0.035
Ever	266	37.5	245	32.2	
Drinking status					
Never	508	71.6	571	75.1	0.120
Ever	202	28.5	189	24.9	
Hypertension					
No	444	62.5	444	73.0	<0.001
Yes	266	37.5	205	27.0	
Diabetes					
No	611	86.1	716	94.2	<0.001
Yes	99	13.9	44	5.8	
Clinical stage					
I	446	62.8			
II	139	19.6			
III	52	7.3			
IV	73	10.3			
Grade					
I	136	19.2			
II	341	48.0			
III	174	24.5			
IV	59	8.3			
Histology					
Clear cell	602	84.8			
Papillary	22	3.1			
Chromophobe	39	5.5			
Unclassified	47	6.6			

* Student's t-test for age and BMI distributions between cases and controls; two-sided χ2-test for others selected variables between cases and controls.

### Genotype and allele frequencies of *AKT1*/*AKT2*/*PTEN*/*MTOR* polymorphisms and RCC risk

The associations between these polymorphisms and RCC risk in the best genetic model are presented in Table 2 and detailed genotype distributions of the polymorphisms are presented Table 3. Genotype frequencies of these 8 SNPs in controls all conformed to Hardy–Weinberg equilibrium. The most significant SNP associated with RCC risk was rs2295080, which is located in the promoter region of *MTOR*. Compared with individuals carrying the rs2295080TT genotype, individuals carrying the TG and TG/GG genotypes were both associated with a reduced risk of RCC (*P* = 0.021, OR = 0.81, 95%CI = 0.68–0.97 and *P* = 0.005, OR = 0.74, 95%CI = 0.59–0.91, respectively). The association between rs2295080 and RCC risk remained significant after adjusting for multiple comparisons (FDR = 0.040). Besides this SNP, the variant homozygote (CC) of another SNP (rs701848), which is located in the 3′UTR region of *PTEN* was also significantly associated with an increased RCC risk (*P* = 0.014, OR = 1.45, 95%CI = 1.08–1.96). However, this association remained only marginally significant after adjusting for multiple comparisons (FDR = 0.056). Since PTEN negatively regulates the mTOR signaling pathway, we then investigated whether there was interaction between the *MTOR* rs2295080 and *PTEN* rs701848 in influencing RCC risk, however, as shown in Table 4, no significant interaction was observed (*P*_interaction_ = 0.118), although individuals with both risk genotypes (rs2295080 TT and rs701848 CC) had a significantly increased RCC risk of 1.72. As the *MTOR* rs2295080 produced the best association signal among the selected polymorphism, we then focused on it in the subsequent functional experiments.

**Table 2:** Primary information for the genotyped SNPs in *AKT1*, *AKT2*, *PTEN* and *MTOR* and their associations with risk for RCC.

Genes	Polymorphisms	Location	Alleles	MAF in database	MAF in controls	*P* for HWE*	Best genetic model		
							*P* *	OR (95CI %)*	FDR†
*AKT1*	rs2494750	5′near gene	G/C	0.267	0.325	0.652	0.181	1.16 (0.93–1.45)	0.290
	rs2498786	5′near gene	G/C	0.242	0.198	0.680	0.583	1.05 (0.84–1.30)	0.666
*AKT2*	rs7254617	5′near gene	G/A	0.144	0.132	0.367	0.135	0.83 (0.64–1.06)	0.270
	rs33933140	3′UTR	A/G	0.465	0.487	0.869	0.729	0.95 (0.70–1.28)	0.729
*PTEN*	rs701848	3′UTR	T/C	0.341	0.404	0.253	0.014	1.45 (1.08–1.96)	0.056
	rs11202607	3′UTR	C/T	0.078	0.109	0.130	0.516	0.92 (0.71–1.19)	0.663
*MTOR*	rs2295080	5′near gene	T/G	0.148	0.241	0.891	**0.005**	**0.74 (0.59–0.91)**	**0.040**
	rs2536	3′UTR	T/C	0.089	0.089	0.353	0.072	0.77 (0.58–1.02)	0.192

* Adjusted for age, sex, smoking, drinking status, diabetes and hypertension in logistic regression model.

† False discovery rate.

SNP: Single-nucleotide polymorphism; MAF: minor allele frequency; 3′UTR: 3′ Untranslated Region; HWE: Hardy-Weinberg Equilibrium; CI: confidence interval; OR: odds ratio. Bold-faced values indicate significant difference after adjusting for multiple comparisons.

**Table 3:** Genotype frequencies of the selected polymorphisms among the cases and controls and their associations with risk of RCC.

Genotypes	Cases, n (%)	Controls, n (%)	*P* *	Adjusted OR (95% CI)*
*AKT1* rs2494750				
GG	300 (42.3)	349 (45.9)		1.00 (reference)
GC	340 (47.9)	328 (43.2)	0.181	1.16 (0.93–1.45)
CC	70 (9.9)	83 (10.9)	0.732	0.94 (0.65–1.35)
GC+CC	410 (57.8)	411 (54.1)	0.298	1.14 (0.90–1.37)
*P _trend_*				0.447
*AKT1* rs2498786				
GG	440 (62.0)	487 (64.1)		1.00 (reference)
*GC*	239 (33.7)	245 (32.2)	0.748	1.03 (0.83–1.30)
*CC*	31 (4.3)	28 (3.7)	0.612	1.15 (0.67–1.97)
*GC+CC*	270 (38.0)	273 (35.9)	0.583	1.05 (0.84–1.30)
*P _trend_*				0.346
*AKT2* rs33933140				
AA	188 (23.6)	199 (26.2)		1.00 (reference)
AG	362 (51.0)	382 (50.3)	0.957	1.01 (0.78–1.39)
GG	160 (22.5)	179 (23.6)	0.729	0.95 (0.70–1.28)
AG+GG	522 (73.5)	561 (73.8)	0.907	0.99 (0.78–1.25)
*P _trend_*				0.720
*AKT2* rs7254617				
GG	564 (79.4)	576 (75.8)		1.00 (reference)
GA	135 (19.0)	168 (11.4)	0.182	0.84 (0.65–1.08)
AA	11 (1.6)	16 (2.1)	0.377	0.71 (0.33–1.57)
GA+AA	146 (20.6)	184 (24.2)	0.135	0.83 (0.64–1.06)
*P _trend_*				0.229
*PTEN* rs11202607				
CC	567 (79.9)	599 (78.8)		1.00 (reference)
CT	138 (19.4)	156 (20.5)	0.516	0.92 (0.71–1.19)
TT	5 (0.7)	5 (0.7)	0.986	1.00 (0.28–3.60)
CT+TT	143 (20.1)	161 (21.2)	0.527	0.92 (0.71–1.19)
*P _trend_*				0.654
*PTEN* rs701848				
TT	222 (31.3)	277 (36.5)		1.00 (reference)
TC	338 (47.6)	351 (46.2)	0.168	1.18 (0.93–1.49)
CC	150 (21.1)	132 (17.3)	**0.014**	**1.45 (1.08–1.96)**
TC+CC	488 (68.7)	483 (63.5)	**0.045**	**1.25 (1.01–1.56)**
*P _trend_*				0.017
*MTOR* rs2295080				
TT	454 (63.9)	438 (57.6)		1.00 (reference)
TG	218 (30.7)	277 (36.5)	**0.021**	**0.81 (0.68–0.97)**
GG	38 (5.4)	45 (5.9)	0.525	0.86 (0.55–1.37)
TG+GG	256 (36.1)	322 (42.4)	**0.005**	**0.74 (0.59–0.91)**
*P _trend_*				0.028
*MTOR rs2536*				
*TT*	607 (85.5)	628 (82.6)		1.00 (reference)
*TC*	99 (13.9)	128 (16.9)	0.074	0.77 (0.58–1.03)
*CC*	4 (0.6)	4 (0.5)	0.811	0.84 (0.19–3.61)
*TC+CC*	103 (14.5)	132 (17.4)	0.072	0.77 (0.58–1.02)
*P _trend_*				0.161

* Adjusted for age, sex, smoking, drinking status, diabetes and hypertension in logistic regression model.

Bold-faced values indicate significant difference at 5% level.

**Table 4:** Interaction analyses of the *MTOR* rs22095080 and *PTEN* rs701848 and risk of RCC.

*MTOR* rs22095080*/PTEN* rs701848*		Cases, n (%)	Controls, n (%)	*P* †	Adjusted OR (95% CI)†
0	GG/TT	12 (1.7)	13 (1.7)	0.009	1.00 (reference)
1	TG/TT or GG/TC	103 (14.5)	130 (17.1)		1.00 (reference)
2	TT/TT or TG/TC or GG/CC	225 (31.7)	289 (38.0)		0.97 (0.71–1.31)
3	TT/TC or TG/CC	269 (37.9)	252 (33.2)		1.33 (0.98–1.81)
4	TT/CC	101 (14.2)	76 (10.0)		1.72 (1.16–2.55)
*P*_trend_					0.001
*P*_interaction_					0.118
Recombined groups*					
0–2		340 (47.9)	432 (56.8)	0.001	1.00 (reference)
3–4		370 (52.1)	328 (43.2)		1.46 (1.18–1.79)

* The number represents the number of risk alleles.

† Adjusted for age, sex, BMI, smoking, drinking status, diabetes and hypertension in logistic regression model. OR: odds ratio; CI: confidence interval.

### Expression of *MTOR* in RCC and the associations between the *MTOR* rs2295080 and *MTOR* expression

**Figure 1:**
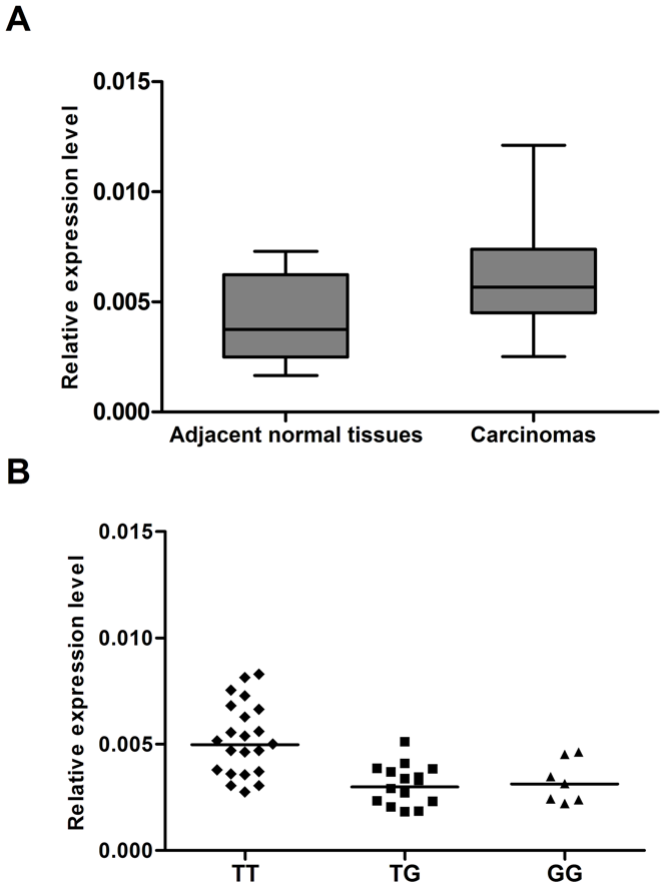
Expression of mTOR in clear cell renal cell carcinomas and adjacent normal renal tissues. (A) Distribution and comparison of *MTOR* expression in carcinomas and adjacent normal tissues (*P* = 0.018). (B) Association between the *MTOR* expression in renal tissues and *MTOR* rs2295080 genotypes. The *MTOR* rs2295080 TT genotype is associated with significantly higher mTOR expression than the *MTOR* rs2295080 TG (*P* = 0.003) and GG (*P* = 0.011) genotypes.

We then explored the expression of *MTOR* in RCC and the associations between the rs2295080 polymorphism and *MTOR* expression in paratumor renal tissues using real-time quantitative RT-PCR. As shown in Figure 1A, the *MTOR* expression level in tumor tissues was significantly higher than that in the adjacent normal tissues (*P* = 0.018). Besides, compared with individuals carrying the TT genotype, individuals carrying the G allele (TG and GG genotypes) had lower levels of *MTOR* expression (*P* = 0.003 and 0.011 for TG *vs.* TT and GG *vs.* TT, respectively) (Fig. 1B). These results suggested that overexpression of *MTOR* may contribute to renal carcinogenesis and that rs2295080, located in the *MTOR* promoter, may be involved in renal carcinogenesis by regulating the transcriptional activity and expression levels of *MTOR*.

### Functional characterization of *MTOR* rs2295080

**Figure 2:**
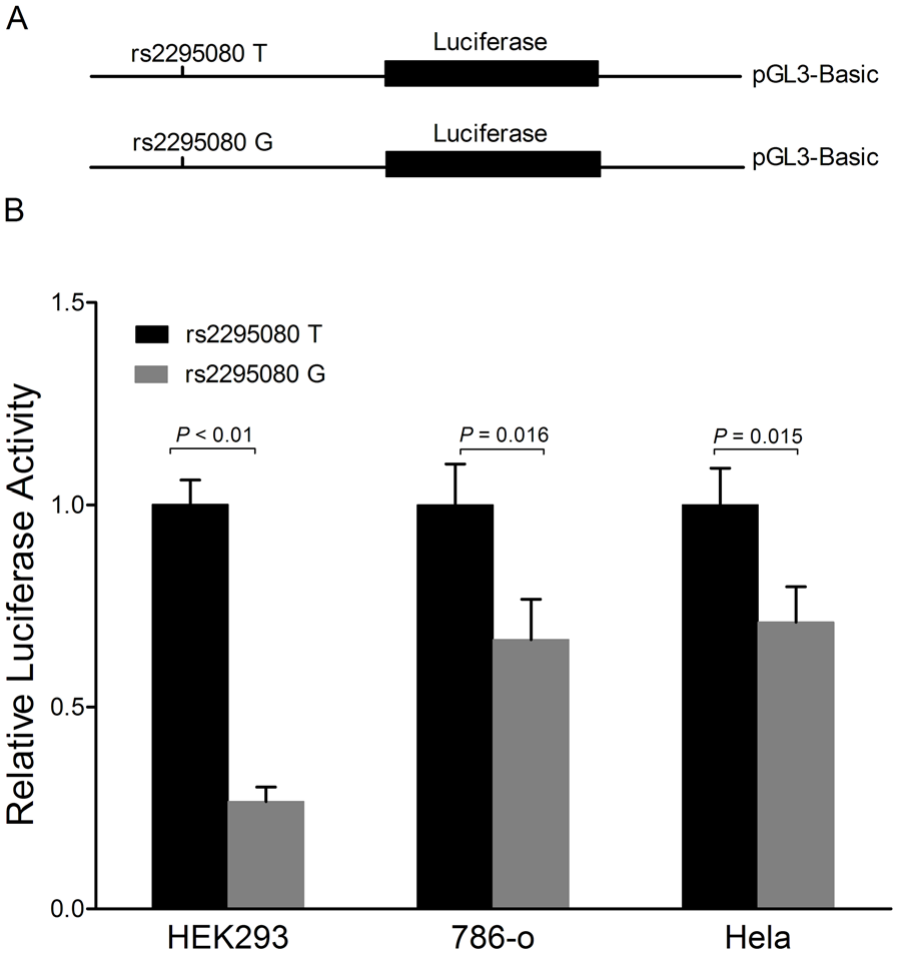
Influence of the *MTOR* rs2295080 polymorphism on the *MTOR* promoter activity. (A) Schematic representation of reporter plasmids containing the *MTOR* rs2295080 T or G allele, which was inserted upstream of the luciferase reporter gene in the pGL3-Basic plasmid. (B) The 2 constructs were transiently transfected into the HEK-293, 786-o and Hela cells, respectively. All of the constructs were cotransfected with pRL-SV40 to standardize the transfection efficiency. Values are means ± SD from more than 3 separate experiments that were each performed in triplicate.

To examine whether the variation is functionally significant by altering the *MTOR* promoter activity, we then generated reporter gene constructs containing either the rs2295080 G or T allele and transfected HEK293, 786-o and HeLa cell lines with the reporter plasmids. As shown in Figure 2, the construct containing the rs2295080G allele drove a significantly lower reporter gene expression compared with that containing the rs2295080T allele in these cell lines. These results indicated that the rs2295080G allele in the promoter region had a reduced transcriptional activity of the *MTOR*.

## Discussion

In the preset study, we investigated the associations between 8 potentially functional polymorphisms in the mTOR signaling pathway-related genes and RCC susceptibility in a Chinese population. Our study suggested that the rs2295080 variant in the promoter region of *MTOR* was associated with a decreased risk of RCC. The association study results of rs2295080 were subsequently confirmed by further functional analysis of the variant. First, we observed that the *MTOR* mRNA level was decreased in individuals who carried the rs2295080 G allele *in vivo*. Then, in the *in vitro* assays, we found that the rs2295080 G allele significantly decreased the transcriptional activity of *MTOR*. These results suggest that the *MTOR* rs2295080 is a functional SNP. To the best of our knowledge, this is the first study to evaluate the role of polymorphisms of mTOR signaling pathway-related genes in the occurrence of RCC.

These findings are biologically plausible, especially in light of the crucial roles of the mTOR pathway in cell death and survival. Over activation of mTOR has been considered a hallmark in RCC, although whether the over activation of mTOR arises from increased protein expression or over phosphorylation of mTOR protein seems vague [20], [21]. Given the important role of mTOR, one would expect that a higher expression level of mTOR total protein may facilitate renal carcinogenesis, which is supported by several studies investigating the expression of *MTOR* in renal cell lines [21] and in nephrectomy RCC specimens [22]. In our study, we also observed that the mRNA level of *MTOR* was significantly higher in RCC tissue than in paratumor renal tissues, which further provided evidence for a causative role of the *MTOR* expression in RCC. Over-expression of *MTOR* has also been suggested to be a poor prognostic factor in several human cancers, including RCC [22], lung cancer [23], breast cancer [24], laryngeal squamous cell carcinoma [25] and biliary tract adenocarcinoma [26]. Considering the role of mTOR in facilitating cancer development and progression, the reduced levels of mTOR owing to the rs2295080 variant in the promoter may decrease cancer susceptibility, which may explain our findings in the association studies.

In addition, the *PTEN* rs701848 polymorphism was marginally associated with an increased RCC risk after adjusting for multiple comparisons. It should be noted that this polymorphism is located in the 3′ UTR region of *PTEN*; therefore it is biologically plausible that this SNP might alter *PTEN* expression by influencing the mRNA stability, and then influence cancer susceptibility. However, the hypothesized function of this SNP still needs to be investigated in future studies. Since the activation of mTOR signaling is negatively regulated by *PTEN*, it would be interesting to see if there is an interaction effect between the *MTOR* rs2295080 and the *PTEN* rs701848 polymorphisms. However, we did not find a significant interaction between these 2 SNPs, although individuals with the risk genotypes of both of the two SNPs (rs2295080 TT and rs701848 CC) had a significantly increased RCC risk of 1.57.

Till now, several molecular target agents, such as the tyrosine kinase inhibitor sunitinib, the VEGFRs inhibitor pazopanib, and the mTOR inhibitors temsirolimus and everolimus, have been approved to treat patients with advanced RCC. Most recently, Xu *et al.* demonstrated that genetic polymorphisms in angiogenesis- and exposure-related genes could predict treatment response to pazopanib monotherapy in patients with RCC [27]. Besides, there is also evidence suggesting that genetic polymorphisms in genes involved in sunitinib pharmacokinetics are associated with progression-free survival (PFS) in mRCC patients treated with sunitinib [28]. It should be noted that the *MTOR* rs2295080 polymorphism has been suggested to be associated with clinical outcomes in esophageal cancer patients treated with chemoradiotherapy [29]. However, there is still a lack of studies investigating the inherited genetic variability in response to the treatment of mTOR inhibitors. Considering the functional role of the rs2295080 polymorphism in modulating the expression of *MTOR*, this polymorphism might be a promising genetic marker to investigate with regard to the prediction of treatment response to temsirolimus or everolimus. However, the lack of available information about these drugs treatments in our RCC patients restricts our study to address this issue; thus, we urge its investigation in other studies that focus on the treatment of RCC patients.

In conclusion, our results suggest that the *MTOR* rs2295080 influences RCC susceptibility in our Chinese population. Our study also highlights that the *MTOR* rs2295080 variant may affect RCC susceptibility by modulating the endogenous *MTOR* expression level. Additionally, the results of our study raise some important questions about, for instance, whether the distinct expression level of *MTOR* induced by the rs2295080 polymorphism will influence the phosphorylation level of mTOR and then alter its downstream signal, and whether this polymorphism has an effect on the RCC prognosis and the response of RCC patients to the treatment with temsirolimus and everolimus. Future studies with more a comprehensive design and additional available information about these drug treatments may help to address these questions.

## Supporting Information

Table S1 **The sequences of the primers and probes used in the present study.**
(DOC)

## References

[1] Ljungberg B, Campbell SC, Cho HY, Jacqmin D, Lee JE, et al (2011) The Epidemiology of Renal Cell Carcinoma. Eur Urol 10.1016/j.eururo.2011.06.049 21741761

[2] Cohen HT, McGovern FJ (2005) Renal-cell carcinoma. N Engl J Med 353: 2477–2490. 16339096 10.1056/NEJMra043172

[3] Chow WH, Dong LM, Devesa SS (2010) Epidemiology and risk factors for kidney cancer. Nat Rev Urol 7: 245–257. 20448658 10.1038/nrurol.2010.46 PMC3012455

[4] Baldewijns MM, van Vlodrop IJ, Vermeulen PB, Soetekouw PM, van Engeland M, et al (2010) VHL and HIF signalling in renal cell carcinogenesis. J Pathol 221: 125–138. 20225241 10.1002/path.2689

[5] Ollerenshaw M, Page T, Hammonds J, Demaine A (2004) Polymorphisms in the hypoxia inducible factor-1alpha gene (HIF1A) are associated with the renal cell carcinoma phenotype. Cancer Genet Cytogenet 153: 122–126. 15350301 10.1016/j.cancergencyto.2004.01.014

[6] Bruyere F, Hovens CM, Marson MN, d'Arcier BF, Costello AJ, et al (2010) VEGF polymorphisms are associated with an increasing risk of developing renal cell carcinoma. J Urol 184: 1273–1278. 20723915 10.1016/j.juro.2010.06.009

[7] Purdue MP, Johansson M, Zelenika D, Toro JR, Scelo G, et al (2011) Genome-wide association study of renal cell carcinoma identifies two susceptibility loci on 2p21 and 11q13.3. Nat Genet 43: 60–65. 21131975 10.1038/ng.723 PMC3049257

[8] Qin C, Cao Q, Ju X, Wang M, Meng X, et al (2012) The polymorphisms in the VHL and HIF1A genes are associated with the prognosis but not the development of renal cell carcinoma. Ann Oncol 23: 981–989. 21778301 10.1093/annonc/mdr325

[9] Cao Q, Qin C, Ju X, Meng X, Wang M, et al (2011) Chromosome 11q13.3 variant modifies renal cell cancer risk in a Chinese population. Mutagenesis 10.1093/mutage/ger085 22131124

[10] Jiang BH, Liu LZ (2009) PI3K/PTEN signaling in angiogenesis and tumorigenesis. Adv Cancer Res 102: 19–65. 19595306 10.1016/S0065-230X(09)02002-8 PMC2933405

[11] Schlessinger J (2000) Cell signaling by receptor tyrosine kinases. Cell 103: 211–225. 11057895 10.1016/s0092-8674(00)00114-8

[12] Stambolic V, Suzuki A, de la Pompa JL, Brothers GM, Mirtsos C, et al (1998) Negative regulation of PKB/Akt-dependent cell survival by the tumor suppressor PTEN. Cell 95: 29–39. 9778245 10.1016/s0092-8674(00)81780-8

[13] Altomare DA, Testa JR (2005) Perturbations of the AKT signaling pathway in human cancer. Oncogene 24: 7455–7464. 16288292 10.1038/sj.onc.1209085

[14] Arcaro A, Guerreiro AS (2007) The phosphoinositide 3-kinase pathway in human cancer: genetic alterations and therapeutic implications. Curr Genomics 8: 271–306. 19384426 10.2174/138920207782446160 PMC2652403

[15] Stiles B, Gilman V, Khanzenzon N, Lesche R, Li A, et al (2002) Essential role of AKT-1/protein kinase B alpha in PTEN-controlled tumorigenesis. Mol Cell Biol 22: 3842–3851. 11997518 10.1128/MCB.22.11.3842-3851.2002 PMC133830

[16] Hudes G, Carducci M, Tomczak P, Dutcher J, Figlin R, et al (2007) Temsirolimus, interferon alfa, or both for advanced renal-cell carcinoma. N Engl J Med 356: 2271–2281. 17538086 10.1056/NEJMoa066838

[17] Hainsworth JD, Spigel DR, Burris HA III, Waterhouse D, Clark BL, et al (2010) Phase II trial of bevacizumab and everolimus in patients with advanced renal cell carcinoma. J Clin Oncol 28: 2131–2136. 20368560 10.1200/JCO.2009.26.3152

[18] Wang W, Pan X, Huo X, Yan F, Wang M, et al (2012) A functional polymorphism C-1310G in the promoter region of Ku70/XRCC6 is associated with risk of renal cell carcinoma. Mol Carcinog 10.1002/mc.21914 22593040

[19] Wang M, Zhang Z, Zhu H, Fu G, Wang S, et al (2008) A novel functional polymorphism C1797G in the MDM2 promoter is associated with risk of bladder cancer in a Chinese population. Clin Cancer Res 14: 3633–3640. 18519798 10.1158/1078-0432.CCR-07-5155

[20] Kruck S, Bedke J, Hennenlotter J, Ohneseit PA, Kuehs U, et al (2010) Activation of mTOR in renal cell carcinoma is due to increased phosphorylation rather than protein overexpression. Oncol Rep 23: 159–163. 19956876

[21] Sourbier C, Lindner V, Lang H, Agouni A, Schordan E, et al (2006) The phosphoinositide 3-kinase/Akt pathway: a new target in human renal cell carcinoma therapy. Cancer Res 66: 5130–5142. 16707436 10.1158/0008-5472.CAN-05-1469

[22] Elfiky AA, Aziz SA, Conrad PJ, Siddiqui S, Hackl W, et al (2011) Characterization and targeting of phosphatidylinositol-3 kinase (PI3K) and mammalian target of rapamycin (mTOR) in renal cell cancer. J Transl Med 9: 133. 21834980 10.1186/1479-5876-9-133 PMC3173341

[23] Gately K, Al-Alao B, Dhillon T, Mauri F, Cuffe S, et al (2012) Overexpression of the mammalian target of rapamycin (mTOR) and angioinvasion are poor prognostic factors in early stage NSCLC: a verification study. Lung Cancer 75: 217–222. 21802763 10.1016/j.lungcan.2011.06.012

[24] Zhou X, Tan M, Stone Hawthorne V, Klos KS, Lan KH, et al (2004) Activation of the Akt/mammalian target of rapamycin/4E-BP1 pathway by ErbB2 overexpression predicts tumor progression in breast cancers. Clin Cancer Res 10: 6779–6788. 15501954 10.1158/1078-0432.CCR-04-0112

[25] Marioni G, Staffieri A, Giacomelli L, Lionello M, Guzzardo V, et al (2011) Mammalian target of rapamycin expression and laryngeal squamous cell carcinoma prognosis: novel preliminary evidence. Histopathology 58: 1148–1156. 21707714 10.1111/j.1365-2559.2011.03864.x

[26] Herberger B, Puhalla H, Lehnert M, Wrba F, Novak S, et al (2007) Activated mammalian target of rapamycin is an adverse prognostic factor in patients with biliary tract adenocarcinoma. Clin Cancer Res 13: 4795–4799. 17699857 10.1158/1078-0432.CCR-07-0738

[27] Xu CF, Bing NX, Ball HA, Rajagopalan D, Sternberg CN, et al (2011) Pazopanib efficacy in renal cell carcinoma: evidence for predictive genetic markers in angiogenesis-related and exposure-related genes. J Clin Oncol 29: 2557–2564. 21576632 10.1200/JCO.2010.32.9110

[28] van der Veldt AA, Eechoute K, Gelderblom H, Gietema J, Guchelaar HJ, et al (2011) Genetic polymorphisms associated with a prolonged progression-free survival in patients with metastatic renal cell cancer treated with sunitinib. Clin Cancer Res 17: 620–629. 21097692 10.1158/1078-0432.CCR-10-1828

[29] Hildebrandt MA, Yang H, Hung MC, Izzo JG, Huang M, et al (2009) Genetic variations in the PI3K/PTEN/AKT/mTOR pathway are associated with clinical outcomes in esophageal cancer patients treated with chemoradiotherapy. J Clin Oncol 27: 857–871. 19164214 10.1200/JCO.2008.17.6297 PMC2738430

