# Supplementary material for: A Functional Variant in the MTOR Promoter Modulates Its Expression and Is Associated with Renal Cell Cancer Risk
Source: PLoS One. 2012 Nov 28;7(11):e50302. doi: 10.1371/journal.pone.0050302 (PMC3508984; doi:10.1371/journal.pone.0050302)
Supplement: Table S1 — The sequences of the primers and probes used in the present study. (DOC) [file pone.0050302.s001.doc]

Table S1- The sequences of the primers and probes used in the present study

| SNPs | primers and probes | Sequence (5’-3’) |
| --- | --- | --- |
| rs2494750 | F | CGGAGGAACTTCTGGCTAGGA |
|  | R | TGGGACACAGACCCCTTTCTC |
|  | G | FAM-CCTGCACTCTCCTGT-MGB |
|  | C | HEX-CCTGCACTGTCCTGT-MGB |
| rs2498786 | F | GGATTCGTCCCTGACCTGTCT |
|  | R | TCAGTTTCCCCGTCTGTAAAGTG |
|  | G | FAM-CTCGCTCTGCCGTGACCCTAGC-MGB |
|  | C | HEX-CTCGCTCTGCGGTGACCCTAGC-MGB |
| rs7254617 | F | GCAGACGTCGGAGTCCTAGTG |
|  | R | TGCGCACATTAGACAACTTTGG |
|  | G | FAM-TGCGTAGCCCCATC-MGB |
|  | A | HEX-TGCGTAGCTCCATC-MGB |
| rs33933140 | F | CAGGCGCCATGCTTCAC |
|  | R | CCAGACTCGCATTTGGAAGAC |
|  | G | HEX-ATTAAAACCTGAGTCTCCAA-MGB |
|  | A | FAM-ATTAAAACCTGAATCTCCAA-MGB |
| rs701848 | F | CATAGTGCTCCCCCGAGTTG |
|  | R | CCGCTTAAAATCGTATGCAGTCT |
|  | T | FAM-ACTAGGGCTTCAATTT-MGB |
|  | C | HEX-ACTAGGGCCTCAATT-MGB |
| rs11202607 | F | TCTTTGTTACAATTTCGGGCAC |
|  | R | AATAATAACAGTG CAAAAGCCCAT |
|  | T | HEX-CGCATATTAAAATGTAACTT-MGB |
|  | C | FAM-CGCATATTAAAACGTAACT-MGB |
| rs2295080 | F | CTTCCCCGCTGTCCTCTAAG |
|  | R | CCATCTTCTCCCTATACCTGTCG |
|  | G | HEX-CTCAGGGCTGGGAA-MGB |
|  | T | FAM-TCAGGGATGGGAAC-MGB |
| rs2536 | F | CATGGTGTCTAGACATGGCTACACT |
|  | R | GAGGTGCTGAACACAGGGAAG |
|  | T | FAM-CTCATAATTTCCAATATGT-MGB |
|  | C | HEX-CTCATAATTCCCAATATG-MGB |
